# The reproductive potential and importance of key management aspects for successful *Calluna vulgaris* rejuvenation on abandoned Continental heaths

**DOI:** 10.1002/ece3.2816

**Published:** 2017-02-27

**Authors:** Katrin Henning, Goddert von Oheimb, Werner Härdtle, Andreas Fichtner, Sabine Tischew

**Affiliations:** ^1^ Department for Nature Conservation and Landscape PlanningAnhalt University of Applied SciencesBernburgGermany; ^2^ Institute of EcologyLeuphana University of LueneburgLueneburgGermany; ^3^ Institute of General Ecology and Environmental ProtectionTU DresdenTharandtGermany

**Keywords:** disturbance, free‐range grazing, germination ability, mowing, seed production, seedling recruitment and survival, soil seed bank

## Abstract

The abandonment of traditional pastoralism as well as the use of heath areas for military purposes has had a major impact on dry heaths in the Continental biogeographical region of Europe, causing severe degradation of its key species *Calluna vulgaris* (L.) HULL. The reproductive potential of this species in a Continental climate is assumed to be low, although there is yet no observational or experimental evidence for this. More knowledge is also needed about cost‐effective and sustainable measures to restore abandoned dry heaths in this biogeographical region, because traditional management options are often too expensive (e.g., sod‐cutting) or restricted due to environmental laws and the danger of unexploded ammunition (e.g., burning). Using as an example an 800 ha Continental heathland in Germany that has been abandoned for about two decades, we studied the reproductive potential (seed production, soil seed bank, and germination ability) of degenerate *C. vulgaris* stands. In addition, we conducted a comprehensive field experiment to test the effects of low‐intensity, year‐round grazing by Heck cattle and Konik horses as well as one‐time mowing and patchy exposure of bare soil on the generative rejuvenation (i.e., recruitment and survival) of degenerate *C. vulgaris* stands over 3 years. We used generalized linear mixed models for statistical analyses. Seed production of degenerate *C. vulgaris* stands was high as well as the germination ability of their seeds, being similar to Atlantic heathlands. However, soil seed‐bank densities were lower than those found in managed or abandoned Atlantic heaths. Overall seedling recruitment in the field was considerably lower in comparison with Atlantic heaths. Low‐intensity grazing or one‐time mowing did not induce a substantial increase in *C. vulgaris* recruitment, whereas an additional one‐time creation of bare soil patches or the one‐time creation of bare soil without subsequent management significantly facilitated seedling recruitment and survival in the first year. However, from the second year on, the positive effect of the creation of bare soil without subsequent management was no longer present. In the third year, survival of juveniles was significantly supported by low‐intensity grazing in combination with shallow soil disturbances as well as in combination with one‐time mowing and shallow soil disturbances, whereas mowing alone resulted in marginally significant lower survival. The extremely low seedling recruitment requires a careful choice of suitable management measures to promote the survival of sufficient numbers of *Calluna* individuals. Therefore, we recommend low‐intensity grazing with free‐ranging robust breeds and the combination of this with one‐time mowing as an effective means of supporting generative rejuvenation of *C. vulgaris* in degraded heaths. However, at the beginning of the restoration process, the creation of bare soil patches for seedling recruitment is crucial. For implementation into practice, we present different strategies to enhance the proportion of bare soil after long‐term abandonment of heaths when traditional management options are no longer feasible.

## 1 Introduction

Heathlands are recognized as being of high conservation value throughout Europe and classified as “habitats of community interest” (Council Directive 92/43/EEC). They are widely distributed across Europe, while dry heaths occurring within the Continental biogeographical region (henceforth referred to as the Continental region) account for 30% of all European dry heaths and cover a habitat area of 907.90 km² (EEA, 2015). Nearly all dry heaths are seminatural, having derived from woodland on dry and acidic sandy soils resulting from a long history of low‐intensity grazing combined with rotational sod‐cutting, mowing, or burning (traditional heathland farming; García et al., 2013; Pywell et al., 1995; Webb, 1998). The removal of the litter and humus layer as a result of these management measures led to nutrient removal and thus to soil impoverishment, which helped to support the generative rejuvenation of its key species *Calluna vulgaris* (L.) HULL (henceforth referred to as *Calluna*). *Calluna* is a light‐demanding, low‐competitive species requiring at least patches of bare soil for the germination of its small seeds (thousand‐seed weight: 0.017 g; Bloemer, 2014) and the survival of its slowly growing juveniles (Iason & Hester, 1993; Quin, Artz, Coupar, Littlewood, & Woodin, 2014). From the middle of the 19th century onwards, traditional heathland practices began to decline due to socioeconomic changes, and large areas of the dry heaths reverted to woodland (Price, 2003). During the 20th century, however, some of these low‐productive areas were used for military training activities, thus maintaining the open nature of the landscape and the *Calluna*‐dominated heaths (Schröder, Balzer, & Ellwanger, 2008; Wanner & Xylander, 2003). More recently, political changes in the former Eastern bloc countries have led to the abandonment of the majority of military training areas in the Continental region (Schumacher & Johst, 2015). In comparison with heaths in the Atlantic biogeographical region (henceforth referred to as the Atlantic region), Continental heaths are less threatened by high air‐borne nitrogen inputs (Erisman, Dammers, Van Damme, Soudzilovskaia, & Schaap, 2015). As a consequence especially of land‐use changes, the conservation status of dry heath habitats is unfavorable‐bad across Europe, with an ongoing deteriorating trend in the Continental region (EEA, 2015). This challenging situation calls for scientifically based restoration schemes to counteract the abandonment of dry heaths in the Continental region, because well‐known traditional measures, such as sod‐cutting and burning, often cannot be applied because they are either too expensive, restricted due to environmental laws or prohibited due to the danger of unexploded ammunition in deeper soil layers as a result of the former military use.

The predominance of degenerate *Calluna* stands and the invasion of competitive grasses are the main degradation processes in abandoned heaths (Britton, Marrs, Carey, & Pakeman, 2000; Dostálek & Frantík, 2015) besides other successional changes such as shrub and tree encroachment (Gimingham, 1992; Newton et al., 2009). Counteracting these processes is difficult in the current socioeconomic situation in Europe and requires the development of cost‐effective restoration approaches, based on a sound understanding of the effects of management on *Calluna* population dynamics and plant community interactions (Bullock & Pakeman, 1996; Bullock et al., 2001; Soons & Bullock, 2008). The essential indicators for predicting and evaluating the rejuvenation process of *Calluna* are the reproductive potential, that is the number of seeds produced, the soil seed bank, and the seed germination ability, as well as the number of recruiting and surviving *Calluna* individuals (i.e., generative rejuvenation). Degenerate *Calluna* stands are characterized by a high proportion of woody or even dead *Calluna* biomass and a reduced proportion of flowering shoots (Gimingham, 1972; Miller & Miles, 1970). Reduced flowering with increasing age might result in a lower annual seed production in comparison with managed heaths where *Calluna* is more vigorous and early life‐history phases are more prominent. This fact in its turn could lead to net losses in the seed bank (Ooi, 2015). In addition, there is evidence that the soil seed bank of *Calluna* is smaller under drier, warmer site conditions in Atlantic heaths (Pakeman, Cummins, Miller, & Roy, 1999; Pywell et al., 2002). Therefore, the soil seed bank might be even smaller in Continental heaths with higher temperatures, less precipitation and drought periods leading to limited seedling recruitment (Ooi, 2015). The less favorable climatic conditions in the Continental region compared to the Atlantic region could also lead to a greater probability of failed recruitment and higher seedling mortality because *Calluna* seedlings and juveniles are highly susceptible to drought (Britton, Marrs, Pakeman, & Carey, 2003; Fagúndez, 2012; Meyer‐Grünefeldt, Calvo, Marcos, von Oheimb, & Härdtle, 2015). Drought events are more common in the Continental than in the Atlantic region, and it is predicted that climate change will lead to an increase in drought periods during the growing season (EEA, 2008; Zacharias, Koppe, & Mücke, 2015). However, no detailed studies are available on the reproductive potential of degenerate *Calluna* in Continental heaths.

Another feature of abandoned heaths with degenerate *Calluna* is a high and dense vegetation structure and a lack of bare soil patches (Mitchell, Rose, & Palmer, 2008; Newton et al., 2009; van Wieren, 2013). In recent years, year‐round grazing by small numbers of free‐ranging robust, large herbivores have replaced the traditional grazing practices in some regions and proved to be a profitable management tool in maintaining dry heaths (Bokdam & Gleichman, 2000; Critchley et al., 2013). Large herbivores have been found to reduce aboveground biomass and create bare soil patches more efficiently than sheep (Mitchell et al., 2008). However, the suitability of this new grazing regime has not yet been investigated for long‐abandoned heaths. At the beginning of the restoration process, degenerate *Calluna* stands might be largely neglected by the grazing animals due to the high proportion of woody biomass. Hence, grazing might not reduce the aboveground biomass and bare soil patch density sufficiently to increase generative *Calluna* rejuvenation. Degraded heaths are often mown before the re‐introduction of grazing management in order to reduce vegetation density and support the vegetative rejuvenation of *Calluna* with resprouting which subsequently enhances the fodder value (Pywell et al., 1995; Webb, 1998). This, in turn, should help to enhance grazing pressure and the creation of bare soil patches. In regions where the re‐introduction of livestock grazing is not viable due to socioeconomic changes, mowing alone is considered to be a practical and affordable substitute (Adamowicz, 2010; Borghesio, 2014; Diemont, de Blust, Heijman, Siepel, & Webb, 2013). However, some studies showed that grazing creates a more open vegetation structure and reduces competing grasses (e.g., *Deschampsia flexuosa*,* Calamagrostis epigejos*,* Molinia caerulea*) more successfully than mowing, due to selective feeding (Borer et al., 2014; Pywell et al., 1995). Thus, the suitability of mowing as a substitute for livestock grazing in heaths remains uncertain.

In order to systematically explore restoration options in abandoned, dry Continental heaths, we conducted a comprehensive 3‐year field experiment to determine the effects of single and combined treatments (low‐intensity grazing, one‐time mowing, one‐time shallow soil disturbance) on the generative rejuvenation of *Calluna* in a large‐scale heath, grazed year‐round by Heck cattle and Konik horses (*Bos taurus* and *Equus ferus caballus*, respectively). In addition, we investigated the seed production, the germination ability of seeds and the soil seed bank of degenerate *Calluna*. Our aim was (1) to close the knowledge gap regarding the reproductive potential of degenerate *Calluna* stands in Continental heaths and (2) to investigate suitable and cost‐efficient management schemes to successfully restore and maintain highly degraded, long‐abandoned Continental heaths in situations where other traditional management measures, such as burning or sod‐cutting, are impossible. We asked the following:

1. Is the reproductive potential of degenerate Continental *Calluna* stands considerably lower than in the Atlantic region?
2. Which of the single and combined treatments best support the generative rejuvenation of *Calluna*?

## 2 Materials and Methods

### 2.1 Study site

The study site is located in the NATURA 2000 site Oranienbaumer Heide (Saxony‐Anhalt, E Germany, 51°46′N, 12°21′E, 70 m a.s.l., 2.683 ha) within the Continental biogeographical region. The mean annual temperature is 9.2°C. The climate is characterized by a mean annual precipitation of about 560 mm (climatologic station: Oranienbaum, period: 1961–1990, DWD, 2015) and arid periods with negative climatic water balances in spring and summer (PIK, 2009). Atmospheric N deposition is less than 10 kg ha^−1^ year^−1^ (Lorenz, Tischew, Osterloh, & Felinks, 2013). The study site is characterized by acidic sandy soils (pH_H2O_: 5.5, base saturation: 61.7%). Grazing is said to have been a major land‐use practice in the area since the fourth century. From 1945 until 1989, the site was used for military training activities, thus maintaining large‐scale open landscapes (John, Lorenz, & Osterloh, 2010). Nearly two decades of abandonment after this (1989 until 2008) led to rapid encroachment by Scots pine (*Pinus sylvestris*) and silver birch (*Betula pendula*). In addition, *C. epigejos*, an indigenous but invasive grass, dominated parts of the area. “European dry heaths” dominated by *Calluna* (habitat code 4030; covering 330 ha) and “Xeric sand calcareous grasslands” (habitat code *6120; 135 ha) are the focus of current nature conservation activities. After pine and birch clearance, a permanent pasture was established at the end of 2008. The pasture encompasses 800 ha and is managed by year‐round, low‐intensity grazing with Heck cattle and Konik horses (stocking rate approximately 0.2 livestock units/ha). In 2012, when this study started, 85% of the *Calluna* stands were still in the degenerate life‐history phase (age: 22 [mean], 33 [maximum] years; K. Henning, unpublished data) and *Calluna* had already died back in some places. Earlier life‐history phases (pioneer phase, building phase, mature phase; Gimingham, 1972) occurred only on patches where grazing pressure was higher due to the proximity of management facilities (mineral licks, watering places) or where *Calluna* was mixed with sandy grassland vegetation and thus more intensively grazed. Besides *Calluna*,* Calamagrostis*,* Festuca ovina*,* Agrostis capilaris, Euphorbia cyparissias,* as well as *Luzula campestris* occurred frequently in the field layer. Rare heathland species such as *Genista pilosa*,* Genista germanica*,* Genista tinctoria*,* Carex ericetorum*,* Rumex acetosella* and *Pilosella officinarum* were less frequently present. The bottom layer of the degraded heathland was characterized by 60%–70% of cryptogams, mainly mosses.

### 2.2 Experimental design and treatments

The experiment was established in areas dominated by heavily degenerate *Calluna*. We used a split‐plot design with six randomly selected, replicated blocks to study the effects of different treatments (Table 1) on seed production, the soil seed bank, and germination ability, as well as on generative rejuvenation of *Calluna* over 3 years (2013–2015).

**Table 1:** Overview of the treatments

Code	Management treatments and dates
C	Control (without management)
G	Grazing: low‐intensity, year‐round grazing with cattle and horses (0.2 livestock units/ha)
M	Mowing once in November 2012
G + M	Grazing and mowing
D	Shallow soil disturbance once in November 2012
G + D	Grazing and soil disturbance
M + D	Mowing and soil disturbance
G + M + D	Grazing and mowing and soil disturbance

**Figure 1:**
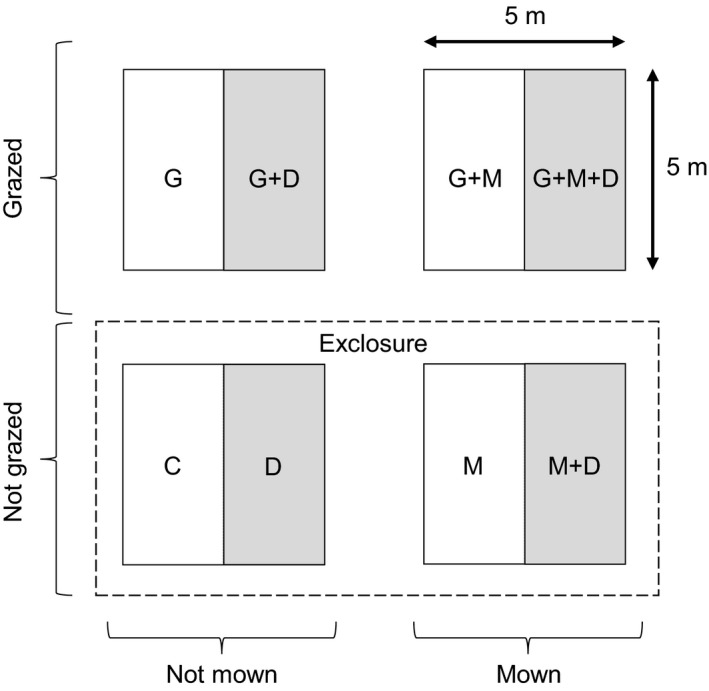
Experimental design: A block consists of four 5 × 5 m plots containing the grazing (G) and mowing (M) treatments and the control (C). Soil disturbances (D treatment) were carried out on one half of each plot (i.e., on subplots of 2.5 × 5 m each, gray shading). Each plot was separated by ca. 15 m buffer strips

A block consisted of four 5 × 5 m plots with ca. 15‐m strips between plots. *Calluna* cover ranged from 70% to 90%. The plots were covered by approximately 70% litter in a 1‐ to 3‐cm‐thick layer over a thin humus layer. Dominating vascular plant species as well as the cryptogam coverage was mentioned above (see study site). Each block consisted of two grazed and two ungrazed plots (exclosures) as well as of two mown and two unmown plots. Grazing (G) and mowing (M) were the main treatments, with a soil disturbance treatment (D) included on half of each plot (i.e., subplots of 2.5 × 5 m each). All possible combinations of these single treatments, including the control (C), resulted in eight different treatments (Figure 1). G treatment plots were grazed as described above (basic management measure in the study area). The ungrazed plots were set up before the implementation of grazing in 2008. For the M treatment, we cut *Calluna* with a brushcutter at a height of 3–10 cm above the ground in November 2012. The clipped material was removed from the plots. A total of 25 small‐scale, shallow soil disturbances (10 × 10 × 3 cm) were randomly distributed over each D treatment subplot. These soil disturbances were supposed to mimic either higher grazing pressure (by imitating the trampling effects of grazers) or deep‐set mowing which exposes bare soil and were manually created by removing the litter and humus layer in November 2012.

### 2.3 Data sampling

#### 2.3.1 Seed production

Seed production was estimated at the plot level by collecting all flowering shoots of *Calluna* individuals on systematically selected 50 × 50 cm quadrats (northern right corner of each plot) at the time of highest seed maturity (end of October 2012) before mowing and soil disturbance were performed. The capsules and seeds of the 24 samples (six blocks with four plots each) were separated from the branches and leaves by hand‐threshing. Because the seeds could not be completely separated from the capsules, the weight of each sample (seeds and capsules) was determined (total weight). A 0.1 g subsample of each sample was randomly selected and the fully developed seeds were counted. The total seed number was extrapolated to the total weight of the sample.

#### 2.3.2 Soil seed bank

Soil samples were collected at the plot level at the end of March 2013, after natural stratification of the seeds had taken place in the field the previous winter. On each plot, 20 randomly positioned soil cores of 10 cm depth were sampled using a soil auger (diameter: 1.4 cm). After removal of the litter layer, the samples were subdivided into two depth layers: 0–2 cm and 2–10 cm. All samples from each plot and depth layer were mixed and spread in a 5‐mm‐thin layer over horticultural perlite and sterile sand in plastic trays (60 × 40 × 6.5 cm), perforated for drainage. Trays were placed in an unheated glasshouse and watered daily for 12 months. Emerging *Calluna* seedlings were counted and removed every 2–3 weeks. Soils were stirred every 8 weeks to expose buried seeds to light. The position of the trays was randomized every second week.

#### 2.3.3 Germination ability (growth chamber experiment)

In order to determine the germination ability of seeds, we randomly took samples from patches in the degenerate life‐history phase (Gimingham, 1972) throughout the study site. Seeds were collected at the end of October 2012 from 50 randomly selected individuals of that life‐history phase. Half of the seeds were stored dry at 5°C for 8 weeks in darkness (cold stratification), while the other half was stored dry at 15°C. In January 2013, six times 100 randomly selected fully developed seeds of each stratification type were sown on moist filter paper in plastic boxes with perforated plastic covers, yielding a total of 6 × 2 = 12 samples each with 100 seeds. The boxes were kept in a growth chamber at a day temperature of 20°C and a night temperature of 10°C with a photoperiod of 12‐hr light and 12‐hr darkness for 90 days. The seeds were regularly watered with tap water. Seeds were counted as germinated when radicles of 0.5 mm were observed. We counted the germinated seeds and rotated the boxes three times a week. The germination rates *t*_10_, *t*_50_, and *t*_90_ were determined as the time in days required to reach 10%, 50%, and 90% of the final germination percentage, respectively.

#### 2.3.4 On‐site generative rejuvenation of *Calluna*

On‐site generative rejuvenation (i.e., recruitment and survival of *Calluna* seedlings/juveniles) was investigated by counting all seedlings at the subplot level in spring 2013 (“recruitment 2013”). The exact location of each seedling was documented on quadrille paper. These individuals were monitored again in autumn 2013 (“survival 2013”). The monitoring of the juveniles was repeated in autumn 2014 and autumn 2015 (“survival 2014” and “survival 2015,” respectively).

### 2.4 Data analysis

We applied generalized linear mixed models to assess the impact of various treatments on the seed production, soil seed bank, and generative rejuvenation of *Calluna*. To account for the hierarchical data structure and overdispersion, we used block as a random factor and a negative binomial error structure with a log‐link function (Zuur, Ienp, Walker, Saveliev, & Smith, 2009). Given the large number of zero counts in the recruitment and survival data, we also accounted for zero inflation in the recruitment (2013) and survival models (2013, 2014, and 2015). Analyses of seedling recruitment in spring 2013 and survival in autumn 2013, 2014, and 2015 were conducted at the subplot level; therefore, we used plot nested within block as random effects in these models.

Due to the sampling date, treatment in the seed production model included only grazing effects, while treatment in the soil seed bank model included grazing and mowing effects (i.e., control, G, M, G + M). To assess the importance of soil layer on the seed number, we also included soil depth (0–2 cm versus 2–10 cm) as a fixed effect in the soil seed bank model. Data from the seed production and the soil seed bank sampling (based on the volume and area of soil sampled in the field and used in the trays) were transformed into seeds per square meter prior to analyses.

The influence of treatments on seedling recruitment and survival included grazing, mowing, and soil disturbance as well as the combined effects of these treatments (see Table 1). Multiple comparisons among treatments were conducted using the Tukey HSD test (*p *< .05).

The germination ability of *Calluna* (growth chamber experiment) was analyzed using percentage data, which were arcsine‐transformed to achieve normality prior to the ANOVAs and *t* tests. Again, Tukey's HSD post hoc test was then run in the case of significant differences.

Data analyses were performed in R 3.2.3 (http://www.R-project.org) using the package glmmADMB (Fournier et al., 2012) and multcomp (Hothorn, Bretz, & Westfall, 2008), and in SigmaPlot 11.0.

## 3 Results

### 3.1 Seed production

The number of seeds per m^2^ produced by degenerate *Calluna* ranged between 139,639 and 760,451 (mean: 345,118 seeds/m^2^, *SD*: 141,205). However, the overall mean did not vary significantly between the G and C plots (*p* = .27).

### 3.2 Soil seed bank

A total of 140,641 and 133,495 seeds germinated from the 0–2 cm and the 2–10 cm soil depth layer samples, respectively, but differences in average seedling emergence between depth layers were not significant (*p *= .22). The highest average seedling emergence was observed in the control plots (4,222 seeds/m^2^, 2–10 cm depth layer) and the lowest seedling density in the G + M plots (1705 seeds/m^2^, 2–10 cm depth layer, Table 2). However, the treatments did not significantly affect mean seedling emergence.

**Table 2:** Density of germinable *Calluna* seeds per m^2^ in the soil seed bank in relation to the soil depth layer (0–2 cm, 2–10 cm depth) and management treatments (grazing and mowing)

Soil layer/management treatment	0–2 cm (*n *=* *6)	2–10 cm (*n *=* *6)
C	2977.4 (363.9)	4222.5 (865.8)
G	2977.4 (697.5)	2733.8 (829.5)
M	2869.1 (675.9)	2463.1 (624.8)
G + M	2896.2 (281.2)	1705.2 (294.1)

C, control; G, grazing; M, mowing; G + M, grazing + mowing. Means ± *SD* are shown.

### 3.3 Germination ability (growth chamber experiment)

**Figure 2:**
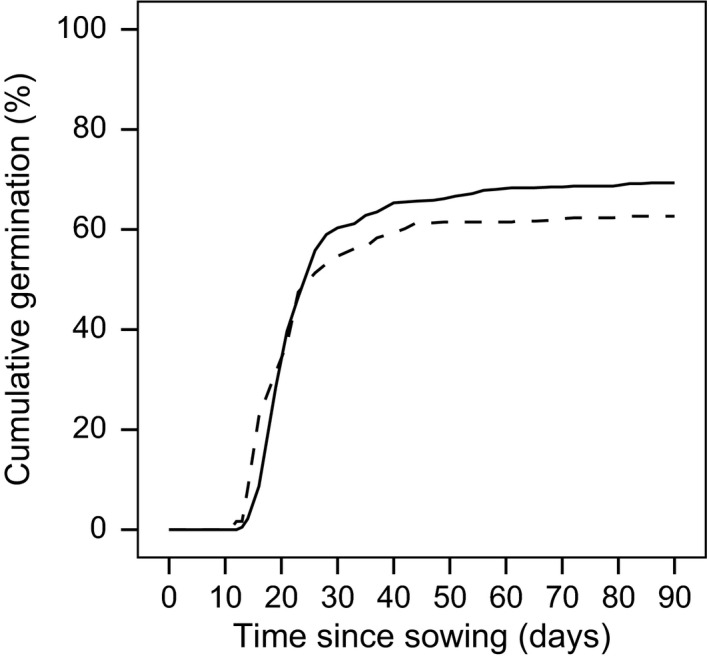
Percentage of cumulative germination over 90 days after sowing seeds collected from *Calluna* in the degenerate life‐history phase. Seeds were subject to two stratification treatments (with cold period: dotted line; without cold period: solid line, *n *=* *6)

The mean germination rates of the growth chamber experiment ranged between 62.7 ± 7.8% (with stratification) and 69.3 ± 8.4% (without stratification; Figure 2). Stratified seeds reached *t*_10_ and *t*_50_, respectively, in a shorter time than seeds without stratification (*p *<* *.001). For stratified seeds, *t*_10_ was achieved within 14 days, *t*_50_ within 19 days and *t*_90_ within 35 days.

### 3.4 Effects of treatments on generative rejuvenation of *Calluna* in the field

In spring 2013, a total of 1,357 *Calluna* seedlings were recorded on all plots with 93.7% surviving until autumn 2013 and 43.6% and 25.8% until autumn 2014 and 2015, respectively. Overall, treatment had a significant effect on seedling recruitment 2013 (*p *<* *.0001) and on survival 2013, 2014, and 2015 (*p *<* *.001 for all years).

**Figure 3:**
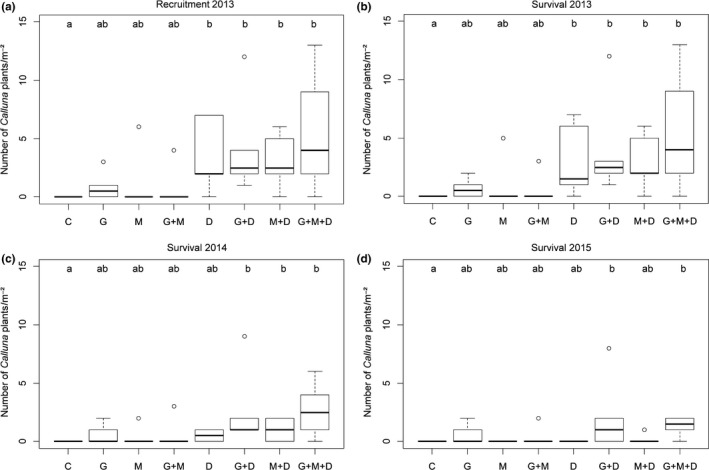
Treatment effects on *Calluna* recruitment in spring 2013 (a) and survival in autumn 2013 (b), 2014 (c) and 2015 (d). Lower case letters indicate significant differences between treatments (Tukey's HSD test; *p *<* *.05). C, control; G, grazing; M, mowing; G + M, grazing + mowing; D, soil disturbance; G + D, grazing + soil disturbance, M + D, mowing + soil disturbance; G + M + D, grazing + mowing + soil disturbance (*n *=* *6)

Seedling recruitment and survival 2013 were highest on subplots with the treatment combination G + M + D (Figure 3a,b). In addition, all other subplots with the D treatment showed a significantly higher seedling recruitment and survival 2013 than the controls (in the order G + D > M + D > D).

For survival 2014 and 2015, the positive effect of the D treatment was no longer present (*p *=* *.16 and .15, respectively), while *Calluna* survival was still higher in G + D, M + D and G + M + D plots than in C plots in 2014 (*p *<* *.001, *p *=* *.01, *p *<* *.001; Figure 3c). Survival was also higher in G + D and G + M + D than C plots in 2015 (*p *<* *.01, *p *<* *.001; Figure 3d). In addition, *Calluna* survival 2015 was marginally lower in M than in G + M + D plots (*p *=* *.08).

## 4 Discussion

### 4.1 Is the reproductive potential of degenerate Continental *Calluna* stands considerably lower than in the Atlantic region?

After nearly 20 years of abandonment, we still found a high annual seed production of degenerate *Calluna* stands in the investigated heathland. Seed production was as high as or even higher than reported for degenerate *Calluna* stands in the Atlantic region (Barclay‐Estrup & Gimingham, 1994; Mallik, Hobbs, & Legg, 1984). However, we are aware that seed production can display wide annual fluctuations depending on weather conditions (Gimingham, 1972; Ooi, 2012). Furthermore, we found high germination rates under controlled conditions (growth chamber) similar to studies from the Atlantic (Pons, 1989; Vera, 1997) and the Mediterranean region (Gonzàilez‐Rabanal & Casal, 1995). In our study, the seeds achieved 90% of their final germination percentage within 35 days, independently of the stratification treatment. This coincides with findings of Gonzàilez‐Rabanal and Casal (1995), whereas Vera (1997) determined a *t*_90_ of 90–120 days. However, the latter study was performed without changes in day and night temperature regime, which can lead to secondary dormancy (Baskin & Baskin, 1998; Pons, 1989). Fluctuating changes in temperature as well as stratification are beneficial to breaking the dormancy of *Calluna* seeds (Måren & Vandvik, 2009; Miller & Cummins, 2001; Pons, 1989) as was also found in our study, where stratification significantly accelerated germination. As temperature fluctuations are more pronounced in the Continental than in the Atlantic region, *Calluna* germination could probably benefit from these climatic conditions on our site.

The long‐term persistent soil seed bank enables *Calluna* to survive under unfavorable environmental conditions and to re‐establish at a later date (Putwain & Gillham, 1990; Bossuyt & Hermy, 2003; Piessens, Honnay, & Hermy, 2005). Contrary to seed‐bank densities of degenerate *Calluna* stands in the Atlantic region, our densities are three times lower than those investigated by Mallik et al. (1984), whereas managed Atlantic heaths show even four to 15 times higher seed densities (Legg, Maltby, & Proctor, 1992; Pywell et al., 2002). Soil seed‐bank densities of our 20‐year abandoned Continental heathland were similar to densities found in heaths that had been afforested by conifers 60 years ago (Pywell et al. (2002) or had undergone succession by birch and pine (Mitchell, Marrs, & Auld, 1998) for 49 years. This was an unexpected finding, because the above‐mentioned abandonment was up to three times longer than in our investigated heath. Considering the climatic gradients, Pakeman et al. (1999) found lower seed‐bank densities for *Calluna* stands on drier, warmer sites in the Atlantic region than on cooler, wetter sites. However, seed‐bank densities in abandoned Atlantic heaths in the south of Great Britain were still five times higher than in our heathland (Mitchell et al., 1998). This confirms our assumption that seed‐bank densities are generally lower under Continental conditions obviously reflecting a limited longevity of *Calluna* seeds in the soil due to differences in soil temperature and moisture (Ooi, 2012).

However, as the seed production of our degenerate *Calluna* stands was still high and the germination of *Calluna* seeds is possible without cold stratification after seed setting in autumn, generative rejuvenation should not generally be restricted.

### 4.2 Which of the single and combined treatments best support the generative rejuvenation of *Calluna*?

#### 4.2.1 Recruitment of *Calluna* seedlings in the first year

The highest seedling recruitment was found on subplots with the treatment combination grazing + one‐time mowing + one‐time soil disturbance. However, all other subplots that included additional experimental, shallow soil disturbances showed higher seedling numbers than subplots without this treatment. These results stress the importance of bare soil patches for successful *Calluna* establishment (de Hullu & Gimingham, 1984; Gimingham, 1972). These open patches may be created, for example, by grazing or other mechanical interventions (Allison & Ausden, 2006; Bullock et al., 2001; Critchley et al., 2013). However, it turned out that low‐intensity grazing alone did not result in a considerable increase in *Calluna* recruitment, showing that the intensity of disturbance is more important than the method of creating bare soil (Mitchell et al., 2008). Thus, additional shallow soil disturbances enhanced the number of microsites for successful *Calluna* recruitment by reducing competition (Pywell et al., 2007) and litter accumulation (de Hullu & Gimingham, 1984) as well as by improving light availability on the ground (Gimingham, 1972). Furthermore, we only found slightly higher seedling numbers on exclusively mown as well as on grazed + mown subplots in comparison to control plots in the first study year, because both measures did not create sufficient amounts of suitable microsites for *Calluna* recruitment.

In contrast to Atlantic heaths (Bokdam & Gleichman, 2000; de Hullu & Gimingham, 1984; Miles, 1974), the overall level of seedling recruitment was considerably lower on our study site despite the observed high‐seed production and germination ability of seeds. This might be related to the drier climatic conditions in the Continental region and the high susceptibility of *Calluna* juveniles to drought (Britton et al., 2003; Fagúndez, 2012; Meyer‐Grünefeldt et al., 2015) leading to a lower establishment success as was observed for drier sites in the Atlantic region by Britton, Carey, Pakeman, and Marrs (2000). Studies from the Continental region found similar low seedling numbers to those observed in our study with no or very low *Calluna* recruitment on unmanaged sites (Sedláková & Chytrý, 1999) and slightly higher numbers on grazed sites (Dostálek & Frantík, 2015).

#### 4.2.2 Survival of *Calluna* juveniles in the third year

Contrary to the seedling recruitment results, after 3 years, the highest number of surviving *Calluna* juveniles was found on grazed + soil disturbed subplots. In addition, all other subplots that included grazing showed higher survival rates than subplots without this treatment. The highest survival rate was observed on grazed + mown subplots (69% survived until 2015), followed by grazing alone and grazing in combination with shallow soil disturbances (65% and 50% survived until 2015, respectively). Thus, our results showed that grazing is crucial for successful survival of *Calluna* over the long term.

Exclusively mown and experimentally disturbed subplots without grazing, as well as the combination of both, showed considerably lower *Calluna* survival after 3 years, because *Calamagrostis* encroached rapidly and suppressed the survival of *Calluna* (K. Henning, unpublished data). Thus, successful generative rejuvenation of *Calluna* cannot be achieved by the one‐time creation of bare soil patches without subsequent management or by an exclusively one‐time mowing. The rapid encroachment of competitive grasses on exclusively mown heaths was also confirmed by other studies from Continental (Sedláková & Chytrý, 1999) and Mediterranean heathlands (Calvo, Alonso, Marcos, & De Luis, 2007). This again stresses the importance of grazing for successful generative rejuvenation of *Calluna* by reducing competition from invasive grasses.

#### 4.2.3 Implications for cost‐efficient restoration schemes in Continental heaths

The restoration of long‐abandoned Continental heaths using cost‐efficient measures, such as low‐intensity grazing with robust cattle and horse breeds, is realistic if sufficient numbers of viable *Calluna* seeds from the seed rain are present and if sufficient patches of bare soil can be created for generative *Calluna* rejuvenation. This means, in practical terms, that grazing and thus trampling intensity must be temporarily and locally enhanced at the beginning of the restoration process. This can be achieved by placing mineral licks as a supplement for the animals' diet or installing temporary fencing in parts of the degraded heathland. Grazing intensity in degraded heathlands can also be enhanced by one‐time mowing as an initial restoration tool to remove the excessive woody or dead biomass, thus leading to increased vegetative rejuvenation of *Calluna* (K. Henning, unpublished data). The regrowth of young shoots subsequently improves the fodder quality of *Calluna* and thereby its attractiveness for the grazing animals. This approach is particularly effective if mosaics of different habitats enable selective foraging. Mowing should be applied rotationally across the heathland to establish a mosaic of diverse structure.

We would like to stress that higher grazing pressure at the beginning of the restoration process should not be achieved by increasing the overall stocking rate in the entire pasture because this would require supplementary feeding and thus a nutritional input into the nutrient‐poor system. If the above‐mentioned options are not feasible, the creation of bare soil can also be fostered by deep‐set mowing which exposes the mineral soil in a patchy way; this type of mowing, however, requires a more or less even terrain and lack of stones.

In all cases, however, low‐intensity grazing is essential for the successful survival and establishment of *Calluna* in the long‐term to prevent the regrowth of highly competing grass species and thus facilitate slowly growing *Calluna* seedlings and juveniles. Furthermore, over the long term, we expect changes in plant species composition, species number, and species diversity due to management, particularly in favor of competitively weak and light needing plants and low‐nutrient indicator species (paper in preparation). In addition, we detected an increase in typical heathland species (paper in preparation). However, it is necessary that seed sources are still present on the site; otherwise the addition of seeds is a key factor in the restoration of species rich heathlands, especially in long‐abandoned heaths.

## Conflict of Interest

None declared.
